# Beyond the buzz: Grounding interoceptive interventions in mechanisms of brain–body coupling

**DOI:** 10.1371/journal.pbio.3003439

**Published:** 2025-11-13

**Authors:** Daniel S. Kluger, Teresa Berther, Martina Saltafossi, Jana Fehring, Joachim Gross

**Affiliations:** Institute for Biomagnetism and Biosignal Analysis, University of Münster, Münster, Germany

## Abstract

The field of interoception research is growing at a rapid pace. This Perspective highlights why establishing both mechanistic insight and construct validity will be critical prerequisites for developing effective body-based health interventions.

## Teaser

The field of interoception research is growing at a rapid pace. We need to establish mechanistic insight into brain–body interactions and validate readouts before we can develop effective body-based health interventions.

The rapidly evolving field of research on interoception, meaning the conscious and unconscious detection of visceral signals, ticks all the boxes of a ‘hot topic’ in modern-day neuroscience. In studying the interplay between brain and body, cardiorespiratory rhythms have taken center stage as modulators of human perception, cognition, and behavior [1]. While the underlying mechanisms are complex and largely unexplored (Box 1), the respiratory rhythm is uniquely under voluntary control and thus gives us a handle for actively manipulating brain–body states [2]. Simply put, these states describe interactions between the brain and the rest of the body across a variety of time scales from the millisecond range (e.g., neural oscillations) up to years (e.g., changes across the life span). Currently, however, the field does not adequately account for these complexities, which can lead to systematic biases in brain–body research, for example, blind spots in our knowledge of hormonal states across the female menstrual cycle have led to marked imbalances in our understanding of female health [3]. After all, the long-term goal of most interoception research is to leverage insights from fundamental research to use in clinical or health applications; so the question becomes, how can we use the current buzz to ensure that interoceptive interventions are standing on solid ground going forward?

**Box 1. Mechanisms of respiration–brain coupling**

The respiratory phase has conclusively been shown to be systematically coupled to the amplitude of both oscillatory [4] and nonoscillatory human brain activity [5,6]. Since these neural responses are indicators of dynamic changes in excitability or arousal states, for example, it comes as no surprise that this coupling also translates to behavior. Fundamental capabilities such as visual perception, emotional processing, memory formation, and motor activity are known to covary with rhythmic respiration [1]. Although we currently have only limited insight into what are likely to be multiple interacting mechanisms that bind neural processing to (cardio-) respiratory physiology, human brain–body neuroscience continues to greatly benefit from extensive animal work on the topic [7]. Therefore, a promising way forward lies in the synthesis of cross-species evidence for the sake of developing grounded computational models to explain and predict brain–body interplay in health and disease.

So far, most health and well-being interventions that involve body work have made use of the respiratory domain in some capacity. A common use of breathing as a means to control physiological states of arousal, for example, is through respiratory heart rate variability [8], by which cardiac signals (such as an elevated heart rate during stress) can be modulated by voluntary changes in respiration. Consequently, clinical interventions for conditions such as panic attack disorder can include interoceptive exposure and different forms of breathing training [9]. Exposure techniques are based on fear extinction principles and expose patients to safe, controlled bodily sensations (such as an elevated heart rate) to mimic panic attack symptoms. The goal is to weaken the negative association between these bodily symptoms and adverse emotional experiences and to enable patients to learn new, nonthreatening associations instead. Breathing training, particularly slow breathing at fewer than 10 breaths per minute, is a less aversive technique of top-down regulation of respiratory rate (compared to exposure therapy). This method can be integrated with biofeedback protocols and has shown benefits in balancing sympathetic and parasympathetic activity in patients with anxiety [9].

Given the potential to turn interoception-related research into usable interventions, the field is at a critical crossroads, where present peak interest could be leveraged to lay the conceptual groundwork for fruitful future avenues. To our minds, there are two key prerequisites for building meaningful interoception interventions. First, we need to develop working models of functional brain–body mechanisms in which any interventions can be grounded. And second, we need to be certain that studies of interoception do indeed measure interoception (and not something else entirely).

The development of meaningful interventions will ideally require mechanistic insight from fundamental brain–body research. As outlined above, breathwork is a central component of most approaches trying to exert control over bodily and mental states. It is important, however, to learn precisely why and how changes in breathing induce changes in other states of the body and the brain. Unfortunately, vaguely-defined constructs like ‘mindfulness’ fall short of providing such much-needed mechanistic insight as they are neither sufficiently falsifiable nor robust enough to withstand rigorous scientific examination. On the contrary, to maximize the efficacy of respiroceptive interventions, we need to quantify the neural correlates of respiratory changes (e.g., with regard to excitability and arousal states) as well as the behavioral consequences they entail. A few studies [5,6] are now beginning to illuminate the neural effects of breathing manipulations (e.g., paced breathing or deep breathing) and inspire speculation regarding human respiration–brain mechanisms, but we are clearly still at the outset. As the groundwork is laid in brain–body research, it will be vitally important that we streamline cross-disciplinary findings by establishing robust and valid measures of interoception.

In light of considerable intra- and inter-personal variability of embodied experience, relying on subjective participant reports alone (“How strongly did you focus on your breathing?”) is clearly neither sensitive nor specific enough to reliably quantify interoceptive processing. To ensure that interoception does not become a mere fuzzy buzzword, our constructs have to pass a certain bar of construct validity: are we really measuring what we think we are? In a rapidly growing field that is trying to establish its own terminology and methodology, the use of conflated theoretical constructs and/or imprecise measures are obvious pitfalls we should be working hard to avoid. Hence, interoceptive paradigms need to be based on carefully defined constructs for subjective measures (such as questionnaires or metacognitive data) and combine them with rigorous objective measures of interoceptive processing. This approach would ideally comprise experimental manipulation of participants’ bodily states and quantification of their sensitivity in accurately recognizing these changes. In consequence, this means letting go of still-popular paradigms such as the heartbeat counting task, which clearly does not provide a sufficiently specific measure of cardiac interoceptive processing [10]. More suitable alternatives, such as the respiratory resistance sensitivity task [11] and the heart rate discrimination task [12], have been developed and validated in the cardiorespiratory domain, and the field ought to continue evolving in this direction.

Overall, we argue that studies of interoceptive processing and interventions resulting therefrom should be grounded in fundamental brain–body neuroscience. To maximize the efficacy of novel interventions, we first have to gain knowledge about their mechanistic underpinnings and continue working toward sensitive and reliable proxies of interoceptive processing. Complementing the development of new constructs and paradigms, much can be gained from methodological advances within the broader field of neuroscience. In trying to comprehensively capture the inherent complexities of brain–body interactions, more and more studies are demonstrating the great value of dense-sampling precision neuroimaging as a means to exploit systematic within-subject variability over different time scales and link, for example, sub-second changes in neural states to monthly variation across the menstrual cycle [13]. Such methodological advances ought to be exploited in a community effort to strengthen the empirical foundation of brain–body research, the field of interoception, and the development of behavioral interventions.
